# Identifikation psychosozial belasteter Familien in pädiatrischen Praxen: Ergebnisse zur Wirksamkeit der PATH(Pediatric Attention To Help)-Intervention

**DOI:** 10.1007/s00103-024-03962-x

**Published:** 2024-10-16

**Authors:** Christian Schlett, Gloria Metzner, Cindy Höhn, Jürgen M. Giesler, Michael Barth, Klaus Kaier, Juliane van Staa, Sabine Horstmann, Susanne Jünemann, Marcus Siebolds, Ilona Renner, Manuela Glattacker

**Affiliations:** 1 Sektion für Versorgungsforschung und Rehabilitationsforschung, Institut für Medizinische Biometrie und Statistik, Medizinische Fakultät, Universitätsklinikum Freiburg, Albert-Ludwigs-Universität Freiburg, Hugstetter Str. 49, 79106 Freiburg, Deutschland; 2 Zentrum für Kinder- und Jugendmedizin, Universitätsklinikum Freiburg, Albert-Ludwigs-Universität Freiburg, Freiburg, Deutschland; 3 Institut für Medizinische Biometrie und Statistik, Medizinische Fakultät, Universitätsklinikum Freiburg, Albert-Ludwigs-Universität Freiburg, Freiburg, Deutschland; 4 Nationales Zentrum Frühe Hilfen, Bundeszentrale für gesundheitliche Aufklärung, Köln, Deutschland; 5 Fachbereich Gesundheitswesen, Katholische Hochschule Nordrhein-Westfalen, Köln, Deutschland

**Keywords:** Frühe Hilfen, Psychosoziale Belastungen, Risikofaktoren, Kinderärzt*innen, Kindergesundheit, Early childhood intervention, Psychosocial burden, Risk factors, Pediatricians, Children’s health

## Abstract

**Hintergrund:**

In Deutschland lebt ca. ein Fünftel der Familien mit kleinen Kindern unter psychosozial belastenden Bedingungen, die die gesunde Entwicklung des Kindes gefährden können. Um die Vermittlung dieser Familien aus der kinderärztlichen Praxis in Angebote der Frühen Hilfen zu verbessern, wurde die PATH-Intervention entwickelt und in Baden-Württemberg implementiert. Ein erster Schritt im Prozess der Vermittlung ist die Identifikation von psychosozial belasteten Familien. Im vorliegenden Beitrag wurde geprüft, ob die PATH-Intervention den Anteil belasteter Familien, die von Praxispädiater*innen als belastet identifiziert werden, erhöht.

**Methode:**

In einer quasiexperimentellen Studie wurden 293 psychosozial belastete Familien untersucht, die von 29 Praxispädiater*innen betreut wurden. Die Interventionsgruppe (IG) waren Familien mit Praxispädiater*innen aus Baden-Württemberg, die an der PATH-Intervention teilgenommen hatten. Die Kontrollgruppe (KG) waren Familien mit Praxispädiater*innen aus Bayern, die nicht an der PATH-Intervention teilgenommen hatten. Mit 10 Praxispädiater*innen der IG und 20 psychosozial belasteten Familien der IG wurden zusätzlich qualitative Telefoninterviews geführt.

**Ergebnisse:**

In der IG wurde ein signifikant höherer Anteil der psychosozial belasteten Familien identifiziert als in der KG. Der Unterschied betrug etwa 20 Prozentpunkte und war unabhängig davon, wie viele Belastungen die Familien aufwiesen.

**Diskussion:**

Die Ergebnisse zeigen, dass die PATH-Intervention die Identifikation von psychosozial belasteten Familien durch Praxispädiater*innen verbessert. Diese verbesserte Identifikation ist eine wichtige Voraussetzung für eine Vermittlung der Familien in passgenaue Unterstützungsangebote wie die der Frühen Hilfen.

**Zusatzmaterial online:**

Zusätzliche Informationen sind in der Online-Version dieses Artikels (10.1007/s00103-024-03962-x) enthalten.

## Abstract (en)

**Background:**

In Germany, about a fifth of families with young children live under psychosocially stressful conditions that can threaten the healthy development of the child. In order to improve the referral of these families from pediatric practices to early childhood intervention services (“Frühe Hilfen”), the PATH intervention was developed and implemented in Baden-Württemberg. The first step in the referral process is to identify psychosocially stressed families. This study investigated whether the PATH intervention increases the proportion of identified families with psychosocial stress by pediatricians.

**Method:**

In a quasi-experimental study, a total of 293 psychosocially stressed families who were cared for by 29 pediatricians from established pediatric practices were examined. The intervention group (IG) consisted of families with pediatricians from Baden-Württemberg who took part in the PATH intervention. The control group (CG) consisted of families with pediatricians from Bavaria who did not take part in the PATH intervention. Additional qualitative telephone interviews were conducted with 10 pediatricians from the IG and 20 families from the IG with psychosocial stress.

**Results:**

A significantly higher proportion of psychosocially stressed families was identified in the IG than in the CG. The difference was about 20 percentage points and was similar regardless of the family’s level of stress.

**Discussion:**

The results show that the PATH intervention improves the identification of psychosocially stressed families by pediatricians. This improved identification of psychosocially stressed families is an important prerequisite for referring families to tailored early childhood intervention services such as those provided by the “Frühe Hilfen”.

## Einleitung

In Deutschland lebt ca. ein Fünftel der Familien mit kleinen Kindern unter psychosozial belastenden Bedingungen [1], die sich schon bei Säuglingen und Kleinkindern negativ auf die Gesundheit und Entwicklung auswirken können [2, 3]. Früh ansetzende präventive Unterstützungsangebote können dazu beitragen, negative Folgen eines Aufwachsens in Belastungslagen abzumildern [4–6]. Dies ist das Ziel der Frühen Hilfen [7]. Die Frühen Hilfen wurden seit 2012 bundesweit flächendeckend ausgebaut und in kommunalen Netzwerken koordiniert [8, 9]. Sie umfassen Angebote, die allen Schwangeren und Eltern mit kleinen Kindern offenstehen. Mit speziellen Angeboten, wie beispielsweise der längerfristig aufsuchenden Begleitung durch Familienhebammen, richten sie sich aber insbesondere an Familien in Belastungslagen.

Gerade Familien mit erhöhtem Unterstützungsbedarf sind mit Präventionsangeboten aber oft nur schwer zu erreichen [10]. Um diesem sogenannten Präventionsdilemma [11] entgegenzuwirken, wurde von Beginn an eine enge Zusammenarbeit zwischen den Frühen Hilfen und der Praxispädiatrie angestrebt [12]. Praxispädiater*innen spielen hierbei eine wichtige Rolle, da die meisten Familien mit kleinen Kindern die kinderärztlichen Früherkennungsuntersuchungen nutzen [13] und Praxispädiater*innen ein hohes Vertrauen entgegengebracht wird. Praxispädiater*innen können das Präventionsdilemma entschärfen, indem sie Familien mit psychosozialen Belastungen identifizieren und bei Bedarf eine Nutzung der Frühen Hilfen empfehlen und anbahnen.

In einer im Jahr 2017 bundesweit durchgeführten repräsentativen Befragung von 815 Praxispädiater*innen zeigte sich allerdings, dass die Überleitung von psychosozial belasteten Familien nicht ausreichend gelingt: Praxispädiater*innen vermittelten demnach nur etwa jede 6. belastete Familie in kommunale Angebote der Frühen Hilfen [14].

Um die Versorgung psychosozial belasteter Familien mit kleinen Kindern zu verbessern, entwickelte das Nationale Zentrum Frühe Hilfen (NZFH) in Zusammenarbeit mit der Kassenärztlichen Vereinigung Baden-Württemberg (KVBW) bereits im Jahr 2010 die sogenannte PATH-Intervention („Pediatric Attention To Help“). Diese wurde vom NZFH aus Mitteln des Bundesministeriums für Familie, Senioren, Frauen und Jugend (BMFSFJ) gefördert. Wie in der Infobox 1 dargestellt, umfasst die PATH-Intervention eine spezielle Schulung der Praxispädiater*innen und die Teilnahme an interdisziplinären Qualitätszirkeln Frühe Hilfen (IQZ).

Die PATH-Intervention ähnelt anderen in Deutschland erprobten Interventionen, die ebenfalls eine Schulung von Pädiater*innen beinhalten und damit auf ein frühzeitiges Erkennen familialer Belastungen und eine Vermittlung in psychosoziale Unterstützungsangebote abzielen [15, 16]. Eine Besonderheit der PATH-Intervention besteht jedoch darin, dass sie neben einer Schulung auch regelmäßig stattfindende IQZ umfasst, in denen sowohl Fälle belasteter Familien als auch Unterstützungsmöglichkeiten durch regionale Angebote besprochen werden. Dadurch soll nicht nur das handlungsrelevante Wissen gesteigert, sondern auch die Vernetzung zwischen den Teilnehmenden unterschiedlicher Sektoren, insbesondere des Gesundheitswesens und der Kinder- und Jugendhilfe, gestärkt werden. Dabei setzt sich die PATH-Intervention folgende Ziele: Praxispädiater*innen sollen psychosozial belastete Familien identifizieren, ihre Unterstützungsbedarfe mit ihnen besprechen, regionale Unterstützungsmöglichkeiten kennen, Eltern über deren Nutzen informieren und sie zur Inanspruchnahme motivieren. Dies soll im Ergebnis dazu beitragen, dass psychosozial belastete Familien vermehrt passende Angebote der Frühen Hilfen in Anspruch nehmen [17].

Inwieweit diese Ziele mit der PATH-Intervention erreicht werden, wurde bislang noch nicht systematisch überprüft. Diese Lücke wurde mit der aus Mitteln des Innovationsfonds geförderten PATH-Evaluation geschlossen [18]. Diese Evaluation prüft die Wirkung der PATH-Intervention auf die Vermittlung von psychosozial belasteten Familien in Angebote der Frühen Hilfen (primärer Endpunkt) sowie die Wirkung auf die (der Vermittlung vorgelagerten) Schritte der Identifikation psychosozial belasteter Familien und deren Informierung und Motivierung zur Inanspruchnahme von Angeboten Früher Hilfen (sekundäre Endpunkte). Zudem untersucht die Evaluation die Akzeptanz der PATH-Intervention bei allen Beteiligten, das Kosten-Effektivitäts-Verhältnis und die Treatment-Integrität.

Im vorliegenden Beitrag wird die Wirkung der PATH-Intervention auf die Identifikation psychosozial belasteter Familien betrachtet. Dieser Aspekt ist von besonderer Relevanz, da das Erkennen einer psychosozialen Belastung nicht nur den ersten Schritt des Vermittlungsprozesses in Angebote der Frühen Hilfen darstellt, sondern darüber hinaus eine wichtige Voraussetzung für jedwedes Eingehen auf psychosoziale Belastungen durch Pädiater*innen ist. Als Hauptfragestellung des vorliegenden Beitrags prüfen wir deshalb, ob die PATH-Intervention die Identifikation psychosozial belasteter Familien durch Praxispädiater*innen verbessert. Unsere Hypothese lautet: Der Anteil identifizierter psychosozial belasteter Familien ist bei Praxispädiater*innen, die an der PATH-Intervention teilgenommen haben (Interventionsgruppe, IG), höher als bei Praxispädiater*innen, die nicht an der PATH-Intervention teilgenommen haben (Kontrollgruppe, KG).

Als ergänzende Fragestellung untersuchen wir, ob die Stärke des Effekts der PATH-Intervention von dem Ausmaß der Belastung einer Familie abhängt. Dies ist anzunehmen, da es für Praxispädiater*innen umso einfacher sein sollte, eine Familie als belastet zu identifizieren, je mehr Risikofaktoren für Belastungen diese aufweist. Je mehr solcher Risikofaktoren eine Familie aufweist, desto weniger sollte es einen Unterschied machen, ob ein*e Praxispädiater*in spezielle Kenntnisse oder Kompetenzen für das Erkennen psychosozial belasteter Familien erworben hat. Unsere Hypothese lautet hier: Der Effekt der PATH-Intervention (d. h. der Unterschied zwischen IG und KG im Erkennen psychosozial belasteter Familien) nimmt mit steigender Anzahl von Risikofaktoren einer Familie ab. Zuletzt analysieren wir als explorative Fragestellung, ob die PATH-Intervention einen Einfluss darauf hat, wie Praxispädiater*innen die psychosoziale Belastung von Familien einschätzen, für die mithilfe des familienseitig eingesetzten Messinstruments kein Risikofaktor ermittelt wurde.

## Methoden

### Studiendesign und Rekrutierung

In einer quasiexperimentellen Studie wurden Familien mit Kindern im Alter von bis zu 3 Jahren, die von Praxispädiater*innen betreut wurden, die an der PATH-Intervention teilgenommen hatten (IG), mit Familien von Praxispädiater*innen verglichen, die nicht an der PATH-Intervention teilgenommen hatten (KG). Für die IG rekrutierte das NZFH, unterstützt von der KVBW, Praxispädiater*innen mit Praxissitz in Baden-Württemberg. Einschlusskriterium waren die Teilnahme an der Interventionsschulung der KVBW oder an einer vergleichbaren Schulung zum Thema Frühe Hilfen bzw. Versorgung psychosozial belasteter Familien sowie die Teilnahme an mindestens 2 IQZ Frühe Hilfen in den letzten 2 Jahren. Für die KG wurden in Zusammenarbeit mit dem Berufsverband der Kinder- und Jugendärzte Bayerns Praxispädiater*innen aus Bayern rekrutiert. Um die Akzeptanz der PATH-Intervention beurteilen zu können [18], wurden mit 10 Praxispädiater*innen der IG und 20 psychosozial belasteten Familien der IG zusätzlich qualitative Interviews geführt. Im vorliegenden Beitrag werden Ergebnisse aus diesen Interviews dargestellt, die Aufschluss über die Identifikation psychosozial belasteter Familien durch Praxispädiater*innen der IG geben und dadurch zur Interpretation der quantitativen Ergebnisse beitragen.

Die Rekrutierung der Familien durch die Praxispädiater*innen fand von Ende Juni 2021 bis Mitte April 2022 statt. Alle teilnehmenden Eltern hatten mit ihrem Kind eine U3–U7 Früherkennungsuntersuchung bei Praxispädiater*innen der IG oder KG besucht (Einschlusskriterium). Im Rahmen dieses Arztbesuchs informierten die Kinderarztpraxen die Familien über die Studie und holten ihre schriftliche Einwilligung ein. Von den Familien, die einer optionalen Teilnahme an einem zusätzlichen Interview zugestimmt hatten, wurden 20 Familien qualitativ befragt. Für diese Interviews kamen nur Familien infrage, für die mithilfe des familienseitig eingesetzten Messinstruments mindestens 2 Risikofaktoren festgestellt wurden. Darüber hinaus mussten die Familien auch arztseitig als belastet eingeschätzt oder es musste arztseitig ein Unterstützungsbedarf bei der Familie gesehen worden sein.

### Quantitative Befragungen

#### Befragungen der Praxispädiater*innen

Die teilnehmenden Praxispädiater*innen gaben nach jeder Früherkennungsuntersuchung mit einer teilnehmenden Familie in einem kurzen Papierfragebogen an, ob sie die Familie als psychosozial belastet einschätzen (Ja/Nein). Dieser Fragebogen war mit der Studien-ID der Familie versehen, wodurch eine Zuordnung zum familienseitigen Fragebogen ermöglicht wurde. Zudem füllten die Praxispädiater*innen nach Abschluss der Rekrutierungsphase einmalig einen Papierfragebogen aus, der Angaben zu ihrer Person (z. B. Alter, Geschlecht und Berufserfahrung) und zur Beschreibung ihrer kinderärztlichen Praxis (z. B. Einzel- oder Gemeinschaftspraxis) erhob.

#### Befragungen der Familien

Mit Eingang der Einwilligungserklärung beim NZFH erhielten die Eltern per E‑Mail einen individualisierten und mit ihrer Studien-ID versehenen Link zur Online-Befragung, die über das webbasierte Softwaretool REDCap [19, 20] umgesetzt wurde. Die meisten Eltern folgten der Einladung und bearbeiteten den Online-Fragebogen (IG = 86 %, KG = 81 %). Der Zeitraum zwischen der Früherkennungsuntersuchung und der Bearbeitung des Fragebogens durch die Familien betrug im Mittel 13 Tage (Standardabweichung (SD) = 11). Der Online-Fragebogen wurde in 4 Sprachen angeboten (Deutsch, Arabisch, Türkisch und Italienisch). Mit Ausnahme einer Familie füllten alle Familien die deutsche Version des Fragebogens aus.

Die psychosoziale Belastung einer Familie wurde in Anlehnung an die NZFH-Studie KiD 0–3 [21, 22] mit dem Psychosozialen Belastungsindex (PSB-Index) erfasst. Dieser Index umfasste in der vorliegenden Studie 23 Indikatoren von Belastungen (z. B. alleinerziehend, beengte Wohnverhältnisse, belastendes Schreiverhalten des Kindes, Zweifel an der erzieherischen Kompetenz; für alle Indikatoren siehe Tabelle Z1 im Online-Material). Wenn die Ausprägungen dieser Indikatoren vorgegebene Grenzwerte überschreiten, werden sie als Risikofaktoren für psychosoziale Belastungen gewertet. Wenn beispielsweise der anhand des PHQ‑4 [23] ermittelte Wert einer Familie ≥ 6 beträgt, stellt dies einen Risikofaktor („yellow flag“) für das Vorliegen einer Depression oder Angststörung und somit einer psychosozialen Belastung dar. Der Gesamtwert des PSB-Index, der PSB-Score, ergibt sich aus der Anzahl der Risikofaktoren einer Familie. Der Anteil fehlender Werte auf Ebene der einzelnen Risikofaktoren und auf Ebene des Gesamtwerts durfte maximal 30 % betragen [24]. Weiterführende Informationen zu den 23 erfassten Risikofaktoren (z. B. deren Items, Berechnung und Grenzwerte) sind im Studienprotokoll [18] ersichtlich.

In Bezug auf den PSB-Score ist zu berücksichtigen, dass ein Wert von 0 nicht belegt, dass eine Familie unbelastet ist, da die Familie Risikofaktoren aufweisen kann, die im PSB-Index nicht erfasst werden. Der PSB-Score stellt somit die Untergrenze der tatsächlich vorliegenden Risikofaktoren einer Familie dar. In Einklang mit vorausgegangenen Arbeiten [15, 25] wurden in der vorliegenden Studie Familien, die laut Selbstauskunft mindestens einen Risikofaktor (d. h. PSB-Score ≥ 1) aufweisen, als mindestens geringfügig psychosozial belastet eingestuft. Wurden diese Familien von ihrer*ihrem Praxispädiater*in ebenfalls als psychosozial belastet eingeschätzt, zählten sie als identifiziert.

### Qualitative Interviews

Für den qualitativen Studienstrang wurden problemzentrierte, teilstrukturierte Telefoninterviews [26, 27] durchgeführt. Hierfür wurde für die Praxispädiater*innen und die Familien jeweils ein spezifischer Leitfaden entwickelt. Die Interviews dauerten durchschnittlich 51 min (Praxispädiater*innen) bzw. 25 min (Familien). Die Telefoninterviews wurden zwischen dem 17.03.2022 und dem 16.06.2022 von einem extern beauftragten unabhängigen Forschungsinstitut durchgeführt.

### Analysen

#### Quantitative Fragebogendaten

**Abb. 1:**
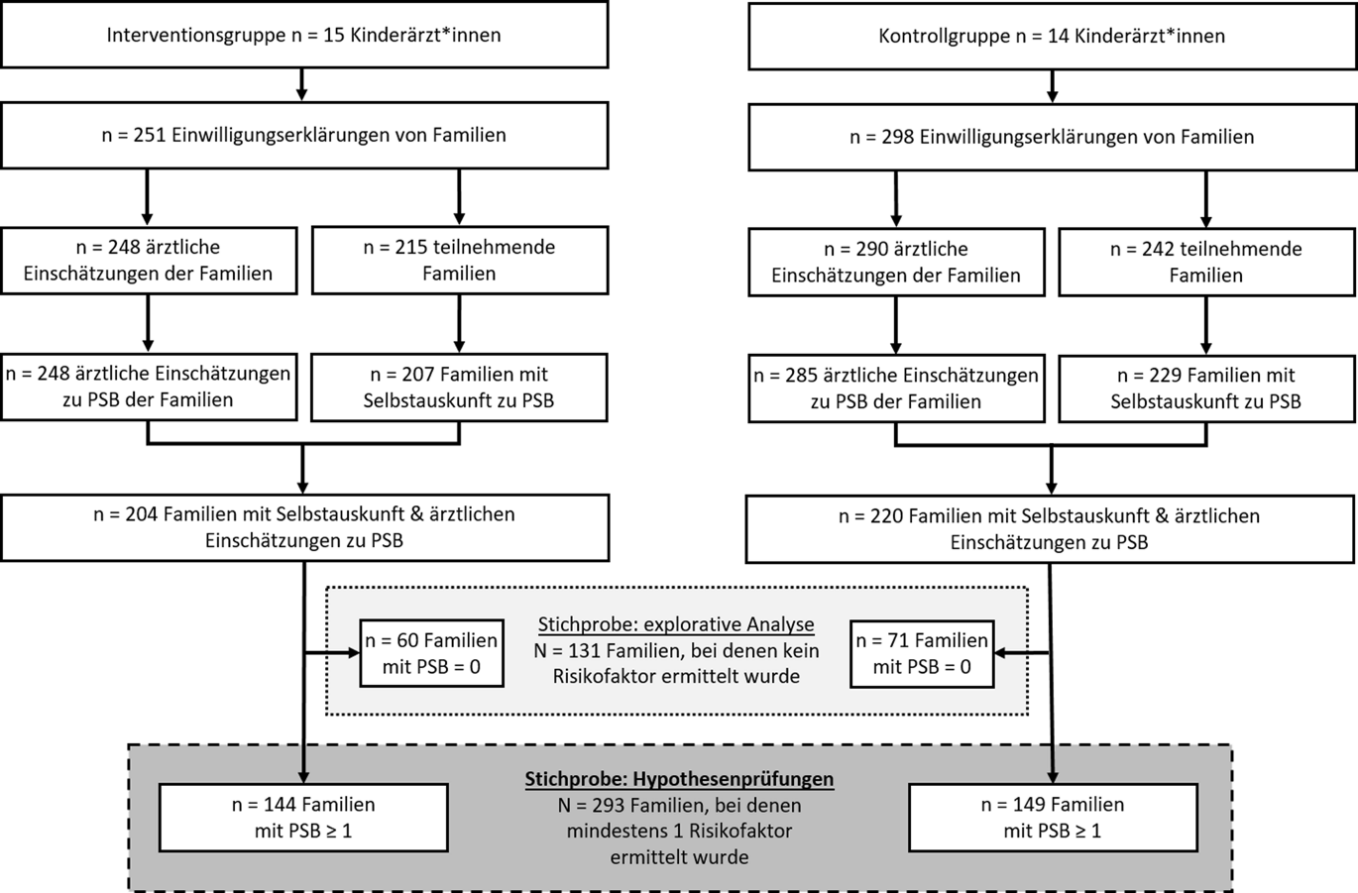
Fluss der Studienteilnehmenden der quantitativen Befragung. Für fast alle Familien, die ihre Einwilligungserklärung zur Studienteilnahme erteilt hatten (*Zeile 2*), lagen Angaben der Praxispädiater*innen zur Einschätzung der PSB der Familien vor (IG = 99 %, KG = 97 %). Am Online-Fragebogen, in dem die PSB als Selbstauskunft erfasst wurde, nahmen die meisten Eltern teil (IG = 86 %, KG = 81 %), jedoch etwas weniger als ärztliche Einschätzungen vorlagen (*Zeile 3*). *N* = Stichprobengröße, *n* = Größe der Teilstichprobe, *PSB* psychosoziale Belastung. (Quelle: eigene Abbildung)

Die statistischen Auswertungen wurden mit Microsoft Excel (Version 16) und IBM SPSS (Version 29.0.0.0) durchgeführt. Vorab wurde der Anteil fehlender Werte geprüft. Hierzu wurde ermittelt, wie viele Familien den Fragebogen bearbeitet, aber nicht genug Items des PSB-Index beantwortet hatten, um einen Gesamtwert zu ermitteln. Dies traf in der IG und der KG jeweils nur auf einen vergleichbar kleinen Anteil der Familien zu (IG = 4 %, KG = 5 %, Abb. 1). Hinsichtlich der ärztlichen Einschätzungen der psychosozialen Belastung der Familien lagen ebenfalls nur sehr wenige fehlende Werte vor (IG = 0 %, KG = 2 %, Abb. 1). Aufgrund der sehr kleinen Anteile fehlender Werte ist von keiner Verzerrung der Hypothesenprüfung durch fehlende Werte auszugehen. Da jeweils mehrere Familien von den gleichen Praxispädiater*innen behandelt wurden, wurden für die Analysen Mehrebenenmodelle angewendet, in denen ein zufälliger Effekt („random intercept“) in das Modell einbezogen wurde. Dieser bildete ab, von welchem*welcher Praxispädiater*in eine Familie behandelt wurde.

In allen Analysen wurde jeweils der Endpunkt untersucht, ob eine Familie von ihrer*ihrem Praxispädiater*in als psychosozial belastet eingeschätzt wurde. Die Analysen zur Hauptfragestellung und zur ergänzenden Fragestellung wurden mit der Stichprobe der Familien durchgeführt, die gemäß dem familienseitigen Fragebogen als belastet eingestuft worden waren. Wurden diese Familien auch von ihren Praxispädiater*innen als belastet eingeschätzt, bedeutete dies, dass sie identifiziert wurden. Die Hypothesenprüfungen erfolgten anhand logistischer Regressionen, bei denen eine Propensity-Score-Adjustierung [28] vorgenommen wurde, um Einflüsse konfundierender Merkmale statistisch zu kontrollieren. Der Propensity-Score wurde aus 6 arztseitigen Merkmalen (Tab. 1), 5 familienseitigen Merkmalen (Tab. 2) und der Zeit zwischen der Früherkennungsuntersuchung und der Beantwortung des Online-Fragebogens (in Tagen) gebildet. Die Hauptanalyse umfasste somit die Prädiktoren Gruppe (IG vs. KG) und Propensity-Score. Zur Beurteilung der Robustheit der Ergebnisse der Hauptanalyse wurde zudem eine Sensitivitätsanalyse ohne Propensity-Score-Adjustierung durchgeführt. Zur Prüfung der Hypothese der ergänzenden Fragestellung, in der postuliert wurde, dass die Stärke des Interventionseffekts vom Ausmaß der Belastung einer Familie abhängt, wurden der PSB-Score und der Interaktionsterm Gruppe x PSB-Score als Prädiktoren ergänzt [29]. Die explorative Fragestellung konnte nicht wie geplant ebenfalls mittels einer logistischen Regression analysiert werden, da in der Stichprobe der Familien mit PSB = 0 in der KG keine Familie von den Ärzt*innen als belastet eingeschätzt wurde und das geplante Verfahren bei einer Häufigkeit von 0 nicht angewendet werden kann. Um diese Fragestellung dennoch zu prüfen, wurde Fishers exakter Test angewendet.

**Tab. 1:** Stichprobe der Praxispädiater*innen (*N* = 29)

Merkmal	IG (*n* = 15)	KG (*n* = 14)
Alter (Jahre), *M (SD)*	52,9 (8,0)	52,7 (10,0)
*Geschlecht, n (%)*		
Weiblich	7 (46,7)	8 (57,1)
Männlich	8 (53,3)	6 (42,9)
Berufserfahrung als Praxispädiater*in (Jahre), *M (SD)*	22,1 (8,9)	22,3 (8,7)
Tätigkeit in niedergelassenen Praxis (Jahre), *M (SD)*	13,7 (8,0)	15,9 (8,7)
*Praxisart*^*a*^*, n (%)*		
Einzelpraxis	6 (40,0)	5 (35,7)
Berufsausübungsgemeinschaft	8 (53,3)	7 (50,0)
Angestellte*r	1 (6,7)	2 (14,3)
*Anzahl Früherkennungsuntersuchungen pro Monat*^*b*^*, M (SD)*	64,8 (34,5)	86,4 (44,5)

*N* = Stichprobengröße, *n* = Größe der Teilstichprobe, *IG* Interventionsgruppe, *KG *Kontrollgruppe, *M* Mittelwert, *SD* Standardabweichung

^a^Aufgrund der geringen Häufigkeiten wurde die Kategorie Angestellte*r für die Bildung des Propensity-Scores zu der Kategorie Berufsausübungsgemeinschaft hinzugefügt, sodass die Variable Praxisart dichotomisiert (Einzel- vs. Gemeinschaftspraxis) in den Propensity-Score einfloss

^b^Anzahl der durch die*den Praxispädiater*in durchgeführten Früherkennungsuntersuchungen während des Rekrutierungszeitraums

**Tab. 2:** Stichprobe der psychosozial belasteten Familien (*N* = 293)

Merkmal	IG (*n* = 144)	KG (*n* = 149)
Alter Kind^a^ (Monate), *M (SD)*	8,7 (8,2)	10,2 (8,5)
*Geschlecht Kind*^*a*^		
Weiblich	76 (52,8)	68 (45,6)
Männlich	68 (47,2)	81 (54,4)
Alter Elternteil (Jahre), *M (SD)*	33,4 (4,6)	32,0 (5,5)
*Geschlecht Elternteil*^*a*^		
Weiblich	133 (92,4)	141 (94,6)
Männlich	11 (7,6)	8 (5,4)
*Migrationsstatus*^*a*^		
Ja	34 (23,8)	30 (20,1)
Nein	109 (76,2)	119 (79,9)
*Höchster Schulabschluss*		
Kein Schulabschluss	0	1 (0,7)
Hauptschulabschluss	7 (4,9)	26 (17,6)
Realschulabschluss	42 (29,2)	50 (33,6)
Fachhochschulreife	16 (11,1)	16 (10,8)
Abitur/Hochschulreife	74 (51,4)	53 (35,8)
Sonstiges	5 (3,5)	2 (1,4)
*Höchster beruflicher Abschluss*		
(Noch) kein beruflicher Abschluss	13 (9,0)	11 (7,5)
Abschluss einer Lehre	29 (20,1)	42 (28,6)
Abschluss an Berufsfachschule	14 (9,7)	21 (14,3)
Meister‑/Technikerausbildung	13 (9,0)	22 (15,0)
Hochschulabschluss	68 (47,2)	48 (32,7)
Sonstiges	7 (4,9)	3 (2,0)
*Nettohaushaltseinkommen*^*a*^		
< 500 €	3 (2,1)	4 (2,7)
500 bis < 1300 €	2 (1,4)	9 (6,1)
1300 bis < 2000 €	17 (11,9)	22 (14,9)
2000 bis < 3000 €	42 (29,4)	52 (35,1)
≥ 3000 €	79 (55,2)	61 (41,2)

*N* = Stichprobengröße, *n* = Größe der Teilstichprobe, *IG* Interventionsgruppe, *KG *Kontrollgruppe, *M* Mittelwert, *SD* Standardabweichung

^a^Variablen, die in den Propensity-Score einbezogen wurden. Aufgrund der geringen Häufigkeiten ging die Variable Nettohaushaltseinkommen dichotomisiert (< 3000 € vs. ≥ 3000 €) in den Propensity-Score ein. Einheit der Angaben = Anzahl (Prozentsatz) der Teilnehmenden, sofern nicht anders angegeben; die Mittelwerte und Standardabweichungen basieren in beiden Gruppen jeweils auf mindestens 98,5 % der Werte (fehlende Werte ≤ 1,5 %)

#### Qualitative Interviewdaten

Die anhand von Audioaufnahmen transkribierten Interviews wurden mit der Methode der inhaltlich strukturierenden qualitativen Inhaltsanalyse ausgewertet [30, 31]. Die Datenanalyse folgte dem systematischen Vorgehen, wie es von Kuckartz [32] vorgeschlagen wird. Die Kategorienbildung erfolgte deduktiv-induktiv. Das anhand der Kategoriensysteme vollständig thematisch strukturierte Material wurde kategorienbasiert, themenspezifisch ausgewertet. Die Ausarbeitung der Kategoriensysteme, die Codierung und die Analyse der Daten erfolgten mithilfe der Software MAXQDA 2022 (Release 22.1.1; [33]). Für die Interrater-Reliabilität ergab sich sowohl für die Interviews mit den Ärzt*innen als auch für die Interviews mit den Familien eine Übereinstimmung zwischen Erst- und Zweitkodiererin von mehr als 70 % (K ≥ 0,72), was als zufriedenstellend bis gut bewertet werden kann [34].

### Registrierung

Vor Beginn der Erhebungsphase wurde die Studie im Deutschen Register Klinischer Studien registriert (DRKS00023461, 03.12.2020). Die Universal Trial Number lautet: U1111-260-6575. Das methodische Vorgehen wurde vor Abschluss der Erhebungsphase in einem Studienprotokoll [18] publiziert.

## Ergebnisse

### Stichprobe

Insgesamt nahmen 29 Praxispädiater*innen aus Baden-Württemberg (IG: *n* = 15) und Bayern (KG: *n* = 14) an der Studie teil. Wie in Tab. 1 zu sehen ist, wiesen die Pädiater*innen der IG und der KG im Mittel sehr ähnliche soziodemografische und berufsbezogene Merkmale auf. Nur in Bezug auf die Anzahl der Früherkennungsuntersuchungen pro Monat gaben die Praxispädiater*innen der IG im Mittel eine niedrigere Anzahl an als die Ärzt*innen der KG. Insgesamt rekrutierten die teilnehmenden Praxispädiater*innen 549 Familien (IG: *n* = 251, KG: *n* = 298). Abb. 1 zeigt den Fluss der Studienteilnehmenden bis zur Stichprobe der psychosozial belasteten Familien, die den Hypothesenprüfungen zugrunde lag. Diese Stichprobe umfasste 293 Familien, für die auf Basis ihrer Selbstauskunft mindestens ein Risikofaktor festgestellt wurde. Tab. 2 zeigt die soziodemografischen Merkmale dieser Familien. Das Alter und das Geschlecht der Kinder, das Alter des antwortenden Elternteils und der Anteil der Familien mit Migrationsstatus fielen in IG und KG ähnlich aus. Allerdings wiesen in der IG mehr Familien einen höheren Schul- und Berufsabschluss und ein höheres Nettohaushaltseinkommen auf als in der KG. Diese Ähnlichkeiten und Unterschiede zwischen der IG und der KG zeigten sich auch in der Stichprobe der Familien, für die kein Risikofaktor ermittelt wurde (siehe Tabelle Z2 im Online-Material).

Von den 10 telefonisch interviewten Praxispädiater*innen waren 5 (50 %) weiblich und 5 (50 %) männlich. Im Mittel arbeiten die Befragten seit 14 Jahren in einer niedergelassenen Praxis. Die 20 Interviews mit Familien wurden alle mit den Müttern der Kinder geführt, mit denen die Früherkennungsuntersuchung aufgesucht wurde (d. h. dem „Zielkind“). Die befragten Mütter waren im Mittel 35 Jahre alt und hatten 2 Kinder. Die Kinder, auf die sich die Mütter in den Interviews bezogen, waren im Durchschnitt etwa 1,5 Jahre alt.

### Hauptanalyse

**Abb. 2:**
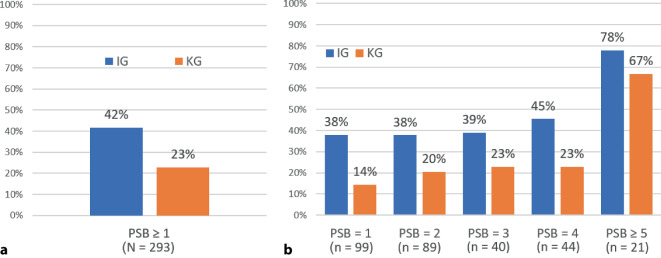
Deskriptive Ergebnisse zum Anteil identifizierter psychosozial belasteter Familien (**a**) insgesamt und (**b**) getrennt nach der Anzahl der psychosozialen Risikofaktoren. *PSB* Psychosoziale Belastung (Anzahl der Risikofaktoren), *N* Stichprobengröße, *n* Größe der Teilstichprobe, *IG* Interventionsgruppe, *KG* Kontrollgruppe. (Quelle: eigene Abbildung)

Zur Prüfung der Hypothese zur Hauptfragestellung wurde zunächst deskriptiv ermittelt, wie viele der gemäß familienseitigem Fragebogen psychosozial belasteten Familien auch von den Praxispädiater*innen der IG und KG als belastet eingeschätzt und somit identifiziert wurden. Wie in Abb. 2a zu sehen ist, traf dies auf 42 % (60 von 144) der Familien der IG zu und auf 23 % (34 von 149) der Familien der KG. Der deskriptive Gruppenunterschied betrug somit 19 Prozentpunkte. Dieser erwartungskonforme Unterschied zwischen der IG und der KG erwies sich in der inferenzstatistischen Prüfung als signifikant (Odds Ratio (OR) = 2,77; *p* = 0,020) und wird durch das Ergebnis der Sensitivitätsanalyse bekräftigt (OR = 2,76; *p* = 0,010). Tab. 3 zeigt die Ergebnisse beider Analysen im Detail.

**Tab. 3:** Vorhersage identifizierter psychosozial belasteter Familien

	Hauptanalyse			Sensitivitätsanalyse			Ergänzende Analyse		
Prädiktor	OR	95 % KI		OR	95 % KI		OR	95 % KI	
Gruppe	2,77*	1,17	6,57	2,76*	1,28	5,96	3,07*	1,29	7,32
Propensity-Score	1,00	0,18	5,58	–	–	–	0,99	0,17	5,62
PSB	–	–	–	–	–	–	1,45*	1,11	1,90
Gruppe x PSB	–	–	–	–	–	–	0,91	0,63	1,33
Pseudo‑R^2^	15,26 %			14,67 %			19,05 %		
ICC	10,43 %			9,86 %			9,45 %		
AIC	1313,16			1312,80			1339,40		

*N* = 293, kontinuierliche Prädiktoren zentriert, *PSB* psychosoziale Belastung (Anzahl der Risikofaktoren), *OR* Odds Ratio, *KI* Konfidenzintervall, Pseudo‑R^2^ bedingt. *ICC* Intraklassenkorrelationskoeffizient der Zugehörigkeit der Familien zu Praxispädiatern, ICC basierend auf dem Nullmodell = 12,91 %, *AIC* Akaike-Informationskriterium

*p < 0,05

Neben der arztseitigen Belastungseinschätzung geben die Ergebnisse der Telefoninterviews zudem Hinweise darauf, dass die Praxispädiater*innen bei der Abschätzung des Unterstützungsbedarfs einer Familie auch die familienseitigen Ressourcen und Lösungsstrategien in den Blick nehmen.

„Also wichtig ist ja immer herauszufinden, wenn die Familie belastet ist, aber sie hat Strategien an der Hand, wie sie mit der Belastung umgehen, dann mache ich mir keine Sorgen“ [#P3].

### Ergänzende Analyse

Zur Prüfung der Hypothese zur ergänzenden Fragestellung wurde ebenfalls zunächst deskriptiv ermittelt, wie viele der psychosozial belasteten Familien in der IG und der KG je nach Anzahl der Risikofaktoren von den Praxispädiater*innen identifiziert wurden (Abb. 2b). Da nur sehr wenige Familien 5 oder mehr Risikofaktoren aufwiesen, wurden diese Familien zu einer Kategorie (≥ 5) zusammengefasst. Wie in Tab. 3 (rechte Spalte) zu sehen ist, zeigte sich für den Prädiktor Gruppe x PSB ein Odds Ratio < 1, das nicht signifikant war (*p* = 0,643), d. h., die Stärke des Interventionseffekts hing nicht signifikant von der Anzahl der Risikofaktoren einer Familie ab. Dieses Ergebnis ist auch in den in Abb. 2b dargestellten deskriptiven Häufigkeiten erkennbar: Der Gruppenunterschied bei Familien mit 1–4 Risikofaktoren betrug im Mittel 20 Prozentpunkte und fiel unabhängig von der Anzahl der Risikofaktoren ähnlich groß aus. Erst bei Familien mit 5 oder mehr Risikofaktoren fiel der Gruppenunterschied mit 11 Prozentpunkten deutlich kleiner aus.

In Einklang mit diesen quantitativen Befunden liefern auch die qualitativen Ergebnisse Hinweise darauf, dass sich die PATH-Intervention unabhängig von der Stärke bzw. der Anzahl und Art der Belastungen der Familien positiv auf das Erkennen von Belastungen auswirken kann. Aus den Interviewergebnissen mit Praxispädiater*innen der IG geht hervor, dass die Teilnahme an den IQZ die Ärzt*innen für psychosoziale Belastungen von Familien zu sensibilisieren scheint. Der Austausch über Fälle (Fallbesprechungen), ein zentrales Element der IQZ, wird als hilfreich für das eigene ärztliche Handeln beschrieben. Dabei ist es insbesondere das Kennenlernen der verschiedenen Blickwinkel und Erfahrungen aus Gesundheitswesen und Kinder- und Jugendhilfe, das als wertvoll wahrgenommen wird.

„… das Erste ist natürlich, dass man aus den Erfahrungen von anderen lernt und dass man beispielhaft dann auch für seine eigenen Patienten an den Beispielen lernt, dass man sensibilisiert wird finde ich ganz wichtig, dass man sensibilisiert zu bestimmten Themen …“ [#P3].

### Explorative Analyse

Zuletzt wurde explorativ überprüft, ob die PATH-Intervention einen Einfluss auf die ärztliche Einschätzung zur psychosozialen Belastung einer Familie hat, für die auf Basis des familienseitigen Fragebogens kein Risikofaktor festgestellt wurde (PSB-Score = 0). Diesbezüglich zeigte sich deskriptiv, dass in der IG 18 % (11 von 60) dieser Familien von ihrer*ihrem Praxispädiater*in als belastet eingeschätzt wurden, wohingegen dies in der KG für keine Familie (0 von 71) zutraf. Im exakten Test nach Fisher (2-seitig) war dieser Unterschied zwischen IG und KG signifikant (*p* < 0,001).

Eine Ursache dafür, dass in der IG ein höherer Anteil von Familien mit einem PSB-Score = 0 als belastet identifiziert wurde, könnte darin liegen, dass Ärzt*innen der IG weitere Arten von Belastungen wahrnehmen, die vom PSB-Index bislang nicht erfasst werden. Hierfür bieten sowohl die Interviews mit den Praxispädiater*innen der IG als auch die Interviews mit den Familien Anhaltspunkte. So werden Belastungen, die sich aufgrund der Betreuung mehrerer Kinder ergeben können, nicht (wie im PSB-Index) an einem sehr jungen Alter der Kinder festgemacht; im Bereich frühkindlicher Regulationsstörungen werden auch die Bereiche Schlafen und Füttern betrachtet (PSB-Index: nur Schreien); es werden Erkrankungen oder Behinderungen auch von Familienangehörigen oder älteren Geschwisterkindern als belastungsrelevant angesehen (PSB-Index: nur vom Zielkind) und schließlich werden auch Belastungen gut situierter Familien in den Blick genommen (für eine ausführliche Darstellung siehe Tabelle Z3 im Online-Material).

„Und dann natürlich frage ich mich ja durch alle Strukturen durch. Wie ist es mit dem Schlafen? Wie ist es mit dem Essen? Wie ist es mit den Geschwistern? Gibt es Geschwisterrivalitäten? Und irgendwann kommen wir schon auf den Punkt, wo wir feststellen, ah, da liegt irgendwas im Argen“ [#P9].

## Diskussion

Die Ergebnisse zur Hauptfragestellung zeigen, dass die PATH-Intervention zu einer signifikanten Verbesserung der Identifikationsrate psychosozial belasteter Familien mit kleinen Kindern führte. Der Anteil der psychosozial belasteten Familien, die von ihren Praxispädiater*innen identifiziert wurden, war in der IG etwa doppelt so groß wie in der KG. Absolut betrachtet konnte durch die PATH-Intervention zusätzlich etwa eine von 5 psychosozial belasteten Familien von Praxispädiater*innen identifiziert werden. Die vorliegenden Ergebnisse belegen somit die Wirksamkeit der PATH-Intervention in Bezug auf diesen wichtigen Teilaspekt im Vermittlungsprozess von der pädiatrischen Praxis in die Frühen Hilfen. In Deutschland wurden zwar bereits ähnliche Interventionen durchgeführt, die als Teilziel ebenfalls die Verbesserung der Identifikation psychosozial belasteter Familien beinhalteten [15, 16]. Inwieweit dieses Teilziel erreicht wurde, wurde bisher aber noch nicht berichtet, sodass eine Einbettung unserer Befunde in diese Studien ausbleiben muss. Die vorliegenden Ergebnisse stimmen jedoch mit Befunden zu dem amerikanischen Programm SEEK [35] überein. Durch dieses Programm, das halbtägige Schulungen für Pädiater*innen zum Erkennen und Ansprechen von psychosozialen Belastungen umfasst, konnte die Häufigkeit des Einsatzes eines Screening-Fragebogens zur Erfassung psychosozialer Belastungen um 18–29 Prozentpunkte gesteigert werden [36]. Die erzielten Steigerungen waren somit ähnlich hoch wie die Steigerungen der Identifikation psychosozial belasteter Familien durch die PATH-Intervention.

Entgegen der Hypothese zur ergänzenden Fragestellung hing die Stärke des Effekts der PATH-Intervention nicht vom Ausmaß der Belastung der Familien ab. Zudem ging aus der Prüfung der explorativen Fragestellung hervor, dass die Praxispädiater*innen der IG einen größeren Anteil der Familien, für die im familienseitigen Fragebogen kein Risikofaktor festgestellt wurde, als psychosozial belastet einschätzten als Pädiater*innen der KG. Dieses Ergebnis kann einerseits als Hinweis interpretiert werden, dass Praxispädiater*innen der IG die psychosoziale Belastung von Familien überschätzen. Andererseits muss ein Wert von 0 im PSB-Index nicht notwendigerweise bedeuten, dass eine Familie unbelastet ist, da die Familie durchaus Belastungen aufweisen kann, die nicht vom PSB-Index erfasst werden. Hierfür sprechen die qualitativen Befunde, die Hinweise auf weitere in der Kinderarztpraxis auffallende Belastungen enthalten, die im PSB-Index nicht berücksichtigt werden. Die qualitativen Ergebnisse weisen zudem darauf hin, dass die PATH-Intervention Praxispädiater*innen für das Erkennen von Belastungen sensibilisieren kann. In jedem Fall führte die PATH-Intervention dazu, dass generell mehr Familien als belastet eingeschätzt wurden, ungeachtet dessen, wie viele Risikofaktoren die Familien aufwiesen. Dieser höhere Anteil identifizierter (auch geringfügig belasteter) Familien ist positiv zu bewerten, da bisher zu wenige belastete Familien von Praxispädiater*innen in Angebote der Frühen Hilfen übermittelt wurden [14] und Praxispädiater*innen nur bei Familien, die sie als belastet einschätzen, weitere Schritte gehen, um die Belastungen der Familien anzusprechen [37] und bei Bedarf eine Vermittlung in Angebote der Frühen Hilfen anzubahnen. Wenn die PATH-Intervention dazu führt, dass Praxispädiater*innen auch einen Anteil von Familien als belastet einschätzen, die möglicherweise nur gering belastet sind, aber aus ärztlicher Sicht dennoch Unterstützungsbedarf haben, muss dies keine negativen Auswirkungen im Sinne einer Fehlversorgung haben, da auch diese Familien von den präventiven Angeboten der Frühen Hilfen profitieren können [38].

Es ist allerdings zu beachten, dass nicht alle psychosozial belasteten Familien externer Unterstützung bedürfen. Ob tatsächlich ein entsprechender Unterstützungsbedarf besteht, hängt auch davon ab, ob die Belastung einer Familie ihre eigenen Ressourcen übersteigt [39]. Außer der Identifikation von Risikofaktoren spielen somit auch die Exploration und Einschätzung der Ressourcen der Familien durch Praxispädiater*innen eine wichtige Rolle für die Ermittlung eines Hilfebedarfs [40]. Die qualitativen Ergebnisse weisen darauf hin, dass Ärzt*innen der IG entsprechend verfahren und prüfen, inwiefern Belastungslagen durch den Einbezug familialer Ressourcen abgemildert werden können.

Einschränkungen der vorliegenden Studie betreffen vor allem die Instrumente, die zur familien- und arztseitigen Erfassung der psychosozialen Belastung eingesetzt wurden. Bei dem PSB-Index handelt es sich um ein Messinstrument, das im Kontext der Frühen Hilfen zur Bestimmung der Prävalenz psychosozialer Belastungen bereits in den großen repräsentativen KiD 0–3 Studien des NZFH [25, 41–43] eingesetzt wurde. Da der PSB-Index nicht alle potenziellen Risikofaktoren abdeckt, kann er zwar psychosozial belastete Familien identifizieren, unbelastete aber nicht. Die Hypothesenprüfung der vorliegenden Studie begrenzt sich deshalb auf die Identifikation von belasteten Familien, d. h. auf eine Überprüfung des Zugewinns an Sensitivität durch die PATH-Intervention. Eine Überprüfung der Identifikation von unbelasteten Familien, d. h. eine Überprüfung des Zugewinns an Spezifität durch die PATH-Intervention, konnte in der vorliegenden Studie nur ansatzweise vorgenommen werden. Den Fokus zunächst auf die Überprüfung des Zugewinns an Sensitivität zu legen, erscheint vor dem Hintergrund angemessen, dass die Identifikation von belasteten Familien für die weitere Versorgung dieser Familien von höherer Bedeutung ist als die Identifikation von unbelasteten Familien.

Weiterhin wurde die psychosoziale Belastung familien- und arztseitig unterschiedlich erhoben. Im Gegensatz zur familienseitigen Messung der psychosozialen Belastung anhand verschiedener Indikatoren beurteilten die Praxispädiater*innen die psychosoziale Belastung der Familien global anhand eines einzigen Items (Schätzen Sie die Familie als psychosozial belastet ein? (Nein/Ja)). Die ärztliche Einschätzung war somit nicht auf die Merkmale begrenzt, auf die sich der PSB-Index stützt, sondern bezog sich vermutlich vielmehr auf einen ganzheitlichen Eindruck der Familie. Die ärztliche Einschätzung konnte zwar mit dem (dichotomisierten) Gesamtwert der familienseitig ermittelten psychosozialen Belastung verglichen werden, es war aber nicht möglich zu prüfen, ob der*die Praxispädiater*in die konkrete(n) Belastung(en) erkannt hat, die sich im PSB-Index abbildete(n). Ein solcher Vergleich wäre aufschlussreich, da damit ergründet werden könnte, ob der durch die PATH-Intervention erhöhte Anteil identifizierter psychosozial belasteter Familien auf ein besseres Erkennen bestimmter Belastungen zurückzuführen ist oder darauf, dass Praxispädiater*innen, die an der PATH-Intervention teilgenommen haben, eine höhere Sensibilität für Belastungen entwickelt haben. Beide Annahmen werden durch Befunde der qualitativen Interviews gestützt.

## Fazit

In der vorliegenden Studie konnte erstmals nachgewiesen werden, dass die PATH-Intervention die Identifikation psychosozial belasteter Familien durch Praxispädiater*innen verbessert. Die Verbesserung betrug etwa 20 Prozentpunkte und war für psychosozial belastete Familien mit bis zu 4 Risikofaktoren, die den Großteil der untersuchten Stichprobe ausmachten, vergleichbar groß. Die Ergebnisse einer explorativen Analyse deuten darauf hin, dass die Teilnahme an der PATH-Intervention dazu führt, dass Praxispädiater*innen generell mehr Familien als belastet identifizieren – möglicherweise (auch) durch eine Wahrnehmung von Risikofaktoren, die mit dem PSB-Index bislang nicht erfasst werden. Mit der Verbesserung der Identifikation psychosozial belasteter Familien schafft die PATH-Intervention eine wichtige Grundlage, um die Vermittlung der betroffenen Familien aus der kinderärztlichen Praxis in passgenaue Unterstützungsangebote wie die der Frühen Hilfen zu verbessern.

### Infobox 1 Komponenten der PATH-Intervention

Die Intervention besteht aus 2 Hauptbestandteilen:

1. Regelmäßig stattfindende interprofessionelle Qualitätszirkel (IQZ), bei denen Fachkräfte des Gesundheitswesens und Mitarbeitende der Kinder- und Jugendhilfe zusammenkommen, um Fälle von belasteten Familien strukturiert aus einer interprofessionellen Perspektive zu besprechen. Außer dem fachlichen Austausch sollen durch die IQZ die interprofessionelle Kooperation und Vernetzung zwischen den Beteiligten verbessert werden.
2. Eine eintägige Schulung für Praxispädiater*innen zu klinischer Fallfindung, die Anregungen zur Exploration und Identifikation von psychosozialen Belastungen enthält. In dieser Schulung werden die Praxispädiater*innen zudem in der Technik des motivierenden Elterngesprächs fortgebildet.

Seit ihrer Einführung im Jahr 2010 wurde die PATH-Intervention in Baden-Württemberg in der Mehrzahl der Städte und Landkreise etabliert (Stand 2023: 9 Stadtkreise und 35 Landkreise).

## Supplementary Information

Indikatoren psychosozialer Belastung und soziodemografische Merkmale der Familien, für die kein Risikofaktor ermittelt wurde
