# Supplementary material for: Identification of psychosocially stressed families by practice pediatricians: Results on the effectiveness of the PATH (Pediatric Attention To Help) intervention
Source: Bundesgesundheitsblatt Gesundheitsforschung Gesundheitsschutz. 2024 Oct 16;67(12):1394–404. [Article in German] doi: 10.1007/s00103-024-03962-x (PMC11615100; doi:10.1007/s00103-024-03962-x)
Supplement: Supplementary file 1 — Indikatoren psychosozialer Belastung und soziodemografische Merkmale der Familien, für die kein Risikofaktor ermittelt wurde [file 103_2024_3962_MOESM1_ESM.docx]

# **Onlinematerial: Psychosoziale Risikofaktoren der Familien**

## **Tabelle Z1**. Psychosoziale Belastungen, die im Psychosozialen Belastungsindex erfasst wurden

| Bereich | Belastung | |
| --- | --- | --- |
| A) Lebenssituation der Familie | | |
| 1. | | Alleinziehend |
| 2. | | Geringe Bildung |
| 3. | | Armut (Bezug staatlicher Leistungen) |
| 4. | | Mehrere kleine Kinder im Haushalt |
| 5. | | Beengte Wohnverhältnisse |
| 6. | | Partnerschaftskonflikte |
| 7. | | Fehlende soziale Unterstützung |
| B) Persönliche Voraussetzungen für die Bewältigung der Fürsorgeanforderungen | | |
| 8. | | Frühe Mutterschaft |
| 9. | | Zweifel an der eigenen erzieherischen Kompetenz |
| 10. | | Negative Kindheitserfahrungen |
| 11. | | Anzeige wegen Gewalt eines Elternteils |
| C) Psychische Gesundheit | | |
| 12. | | Impulsivität/Ärgerneigung |
| 13. | | Despression/Angst |
| 14. | | Hinweise auf Substanzmissbrauch |
| D) Verhalten während der Schwangerschaft und Haltung gegenüber dem Kind | | |
| 15. | | Ungeplante Schwangerschaft |
| 16. | | Unregelmäßige Schwangerschaftsvorsorgeuntersuchungen |
| 17. | | Negative Attribution (des Verhaltens des Kindes) |
| E) Besondere Fürsorgeanforderungen durch das Kind | | |
| 18. | | Perinatale Probleme (Frühgeburt oder geringes Geburtsgewicht) |
| 19. | | Negative Affektivität des Kindes (häufige Wut-/Trotzanfälle) |
| 20. | | Belastendes Schreiverhalten |
| 21. | | Behinderung oder Erkrankung des Kindes |
| F) Problematisches Fürsorgeverhalten | | |
| 22. | | Bindung/Schwierigkeiten sich in das Kind einzufühlen |
| 23. | | Tendenz zur Überreaktion (härteres Anfassen) |

Die psychosoziale Belastung von Familien mit kleinen Kindern wurde in der PATH-Studie mit dem Psychosozialen Belastungsindex (PSB) erfasst, der von der Bezugsperson ausgefüllt wurde. Der PSB umfasst 23 Indikatoren, die je nach Ausprägung psychosoziale Belastungen indizieren. Die einzelnen Belastungsindikatoren können sechs Bereichen (A-F) zugeordnet werden.

## **Tabelle Z2**. Stichprobe der Familien, für die kein Risikofaktor ermittelt wurde (N = 131)

| Merkmal | IG (*n* = 60) | KG (*n* = 71) |
| --- | --- | --- |
| Alter Kind^a^ (Monate), *M (SD)* | 9,4 (7,2) | 7,7 (6,5) |
| Geschlecht Kind^a^ |  |  |
| weiblich | 26 (44,1) | 31 (43,7) |
| männlich | 33 (55,9) | 40 (56,3) |
| Alter Elternteil (Jahre), *M (SD)* | 33,4 (4,0) | 31,9 (3,4) |
| Geschlecht Elternteil^a^ |  |  |
| weiblich | 57 (95,0) | 69 (97,2) |
| männlich | 3 (5,0) | 2 (2,8) |
| Migrationsstatus^a^ |  |  |
| ja | 10 (16,7) | 6 (8,5) |
| nein | 50 (83,3) | 65 (91,5) |
| Höchster Schulabschluss |  |  |
| kein Schulabschluss | 0 | 0 |
| Hauptschulabschluss | 1 (1,7) | 2 (2,8) |
| Realschulabschluss | 15 (25,0) | 31 (43,7) |
| Fachhochschulreife | 6 (10,0) | 8 (11,3) |
| Abitur/Hochschulreife | 38 (63,3) | 30 (42,3) |
| Sonstiges | 0 | 0 |
| Höchster beruflicher Abschluss |  |  |
| (noch) kein beruflicher Abschluss | 0 | 0 |
| Abschluss einer Lehre | 14 (23,3) | 25 (35,2) |
| Abschluss an Berufsfachschule | 5 (8,3) | 8 (11,3) |
| Meister-/Technikerausbildung | 3 (5,0) | 8 (11,3) |
| Hochschulabschluss | 36 (60,0) | 29 (40,8) |
| Sonstiges | 2 (3,3) | 1 (1,4) |
| Nettohaushaltseinkommen^a^ |  |  |
| < 500 Euro | 0 | 0 |
| 500 bis < 1300 Euro | 0 | 0 |
| 1300 bis < 2000 Euro | 2 (3,4) | 3 (4,3) |
| 2000 bis < 3000 Euro | 8 (13,8) | 20 (28,6) |
| ≥ 3000 Euro | 48 (82,8) | 47 (67,1) |

^a^ Variablen, die in den Propensity-Score einbezogen wurden. Aufgrund der geringen Häufig­keiten ging die Variable Nettohaushaltseinkommen dichotomisiert (< 3000 Euro vs. ≥ 3000 Euro) in den Propensity-Score ein. Einheit der Angaben = Anzahl (Prozentsatz) der Teilnehmenden, sofern nicht anders angegeben; die Mittelwerte und Standardabweichungen basieren in beiden Gruppen jeweils auf 100% der Werte (d. h. es lagen keine fehlende Werte vor), N = Stichproben­größe, n = Größe der Teilstichprobe, IG = Interventionsgruppe, KG = Kontrollgruppe, M = Mittelwert, SD = Standardabweichung.

## **Tabelle Z3**. Psychosoziale Belastungen, die über die Indikatoren des Psychosozialen Belastungsindex hinaus von Praxispädiater*innen erfragt wurden

| Belastung | Praxispädiater*innen | Familien |
| --- | --- | --- |
|  | Ankerzitate | |
| Geschwisterkinder höheren Alters bzw. unterschiedlicher Altersgruppen (auch Geschwisterrivalitäten)  [PSB-Index: nur mind. drei sehr kleine Kinder (bis zwei Jahre) als Risikofaktor] | *Ist es das erste Kind oder sprechen wir von einer großen Familie, wo es auch unter den Nägeln brennt, weil die fünf Kinder haben?* [#P1] | *Also wenn man drei Kinder hat und ein Säugling dabeihat und die anderen zwei hat, also ich hätte wahrscheinlich wirklich ab und zu mal Hilfe gebraucht […], aber eben nicht wegen dem Kleinen, sondern wegen den größeren Kindern.* [#F10] |
|  |  |  |
| Frühkindliche Regulationsstörungen: Schlafen und Füttern  [PSB-Index: nur Schreien als Risikofaktor] | *Dann auch eben dieser systemische Blick: Was belastet? Also Thema Schlaf, Thema Ernährung, Thema kindliche Unruhe im Säuglingsalter. […] um dann eben zu sehen: Habe ich es mit einer Belastungssituation zu tun?* [#P5] | *Ja, wir haben das Thema angesprochen aktiv, weil wir uns einfach nicht mehr zu helfen wussten. […] Und weil wir auch nicht das Gefühl hatten, dass er Schmerzen hat. Sondern dass es einfach wirklich eine pure Schlaflosigkeit war.* [#F18] |
|  |  |  |
| Erkrankungen / Behinderungen von Familienangehörigen oder älteren Kindern  [PSB-Index: nur Erkrankung / Behinderung des Zielkindes als Risikofaktor] | *Zum Beispiel hatten wir einmal einen schizophrenen Vater, was ein sehr schwieriges Problem dann für die Kindsbetreuung oder überhaupt für das Kind darstellte mit allen Folgen.* [#P9] | *Also, unser ältester Sohn hat eine Enkopresis und Enuresis […]. Der ist im Februar 7 geworden […]. Ich brauche Ihnen gar nicht sagen – das ist natürlich eine Wahnsinnsbelastung.* [#F6] |
|  |  |  |
| Belastungen gut situierter Familien  [PSB-Index: nicht enthalten, nur Armut als Risikofaktor] | *[…] und beim dritten Kind sind sie nach meinem Gefühl dann angestrengt, weil sie häufig andere (...) Aufgaben im Leben haben. Die Belastung durch die Geschwisterkinder, durch Familienorganisation […] Dann ziehen viele um oder sie bauen was um, sind dadurch noch zusätzlich belastet. Also das sind solche, sage ich jetzt mal, Luxusprobleme, die aber trotzdem die Familien belasten.* [#P7] | *Ich glaube, sie haben schon viele, wo sie sich um viel grundsätzlichere existenzielle Sorgen und Probleme der Eltern Gedanken machen müssen. Und ich bin mir manchmal nicht sicher, ob man dann da auch so den Blick hat für uns. Wo man vielleicht auch sagen kann, wir haben auch große Schwierigkeiten irgendwie […].* [#F6] |
